# Classifying maternal deaths in Suriname using WHO ICD-MM: different interpretation by Physicians, National and International Maternal Death Review Committees

**DOI:** 10.1186/s12978-020-01051-1

**Published:** 2021-02-19

**Authors:** Lachmi R. Kodan, Kim J. C. Verschueren, Affette M. McCaw-Binns, Ray Tjon Kon Fat, Joyce L. Browne, Marcus J. Rijken, Kitty W. M. Bloemenkamp

**Affiliations:** 1 Department of Obstetrics and Gynecology, Academic Hospital Paramaribo (AZP), Paramaribo, Suriname; 2 Division Women and Baby, Department of Obstetrics, Wilhelmina’s Children Hospital, University Medical Center Utrecht, Utrecht University, Utrecht, The Netherlands; 3 Julius Global Health, The Julius Center for Health Sciences and Primary Care, University Medical Center Utrecht, Utrecht University, Utrecht, The Netherlands; 4 Department of Obstetrics and Gynecology, St. Vincentius Hospital, Paramaribo, Suriname; 5 Department of Community Health and Psychiatry, University of the West Indies, Kingston, Jamaica

**Keywords:** Inter-rater reliability, Chain of events, WHO ICD-MM, Classification, Pregnancy-related deaths, Underlying cause, Maternal death review committees

## Abstract

**Plain English summary:**

The World Health Organization (WHO) provides a framework (ICD-MM) to classify pregnancy-related deaths systematically, which enables global comparison among countries. We compared the classification of pregnancy-related deaths in Suriname by the attending physician and by the national maternal death review (MDR) committee and among the MDR committees of Suriname, Jamaica and the Netherlands. There were 89 possible pregnancy-related deaths in Suriname between 2010 and 2014. Nearly half (47%) were classified differently by the Surinamese MDR committee as compared to the classification of the attending physicians. All three MDR committees agreed that 18% (n = 16/89) of the cases were no maternal deaths. Out of the remaining 73 cases, there was disagreement regarding whether 15% (n = 11) were maternal deaths. The Surinamese and Jamaican MDR committees achieved greater consensus in classification than the Surinamese and the Netherlands MDR committees. The Netherlands MDR committee classified more deaths as unspecified than Surinamese and the Jamaican MDR committees. Underlying causes that achieved a high level of agreement among the three committees were abortive outcomes and obstetric hemorrhage, while little agreement was reported for unspecified and other direct causes.

The issues encountered during maternal death classification using the ICD-MM guidelines included classification of suicide during early pregnancy; when to assume pregnancy without objective evidence; how to count maternal deaths occurring outside the country of residence; the relevance of direct or indirect cause attribution; and how to select the underlying cause when direct and indirect conditions or multiple comorbidities co-occur. Addressing these classification barriers in future revisions of the ICD-MM guidelines could enhance the feasibility of maternal death classification and facilitate global comparison.

**Background:**

Insight into the underlying causes of pregnancy-related deaths is essential to develop policies to avert preventable deaths. The WHO International Classification of Diseases-Maternal Mortality (ICD-MM) guidelines provide a framework to standardize maternal death classifications and enable comparison in and among countries over time. However, despite the implementation of these guidelines, differences in classification remain. We evaluated consensus on maternal death classification using the ICD-MM guidelines.

**Methods:**

The classification of pregnancy-related deaths in Suriname during 2010–2014 was compared in the country (between the attending physician and the national maternal death review (MDR) committee), and among the MDR committees from Suriname, Jamaica and the Netherlands. All reviewers applied the ICD-MM guidelines. The inter-rater reliability (Fleiss kappa [κ]) was used to measure agreement.

**Results:**

Out of the 89 cases certified by attending physicians, 47% (n = 42) were classified differently by the Surinamese MDR committee. The three MDR committees agreed that 18% (n = 16/89) of these cases were no maternal deaths, and, therefore, excluded from further analyses. However, opinions differed whether 15% (n = 11) of the remaining 73 cases were maternal deaths. The MDR committees achieved moderate agreement classifying the deaths into type (direct, indirect and unspecified) (κ = 0.53) and underlying cause group (κ = 0.52). The Netherlands MDR committee classified more maternal deaths as unspecified (19%), than the Jamaican (7%) and Surinamese (4%) committees did. The mutual agreement between the Surinamese and Jamaican MDR committees (κ = 0.69 vs κ = 0.63) was better than between the Surinamese and the Netherlands MDR committees (κ = 0.48 vs κ = 0.49) for classification into type and underlying cause group, respectively. Agreement on the underlying cause category was excellent for abortive outcomes (κ = 0.85) and obstetric hemorrhage (κ = 0.74) and fair for unspecified (κ = 0.29) and other direct causes (κ = 0.32).

**Conclusions:**

Maternal death classification differs in Suriname and among MDR committees from different countries, despite using the ICD-MM guidelines on similar cases. Specific challenges in applying these guidelines included attribution of underlying cause when comorbidities occurred, the inclusion of deaths from suicides, and maternal deaths that occurred outside the country of residence.

## Background

The maternal mortality ratio (MMR) is a robust indicator of health care quality, inequality and inequity in and among countries [1]. Most maternal deaths are preventable in low, middle and high resource settings, as was the case for 47% of maternal deaths in Suriname between 2010 and 2014 [2, 3]. To develop prevention strategies, accurate data on the number of maternal deaths and insight into underlying causes are essential [2, 4, 5]. However, the assignment of a reliable underlying cause of death and the subsequent classification can be a challenge [6].

The World Health Organization (WHO) aimed to create uniform maternal death classification guidelines to enhance usability, improve comparability and decrease coding errors [7–9]. Therefore, the WHO launched the International Classification of Diseases-Maternal Mortality (ICD-MM) in 2012, an application of International Classification of Diseases-10^th^ edition (ICD-10) to classify deaths during pregnancy, childbirth and the puerperium [7].

Difficulties in attributing the underlying causes can result in inconsistencies in classification in and among countries, despite using the ICD-MM guidelines [8, 10]. When a European expert panel reviewed pregnancy-related deaths across 13 European countries, they identified 14% more maternal deaths than what the national registries of the individual countries included [11]. Classification is especially complicated when comorbidities occur, and the start of the chain of events resulting in maternal death has to be determined [10]. Consequently, underlying cause attribution may vary, or the causes are unknown or unclear, resulting in underreporting. This is not only an issue in low- and middle-income countries but also in high-income countries and was reported by various Maternal Death Review (MDR) committees, including those from Suriname, Jamaica and the Netherlands [3, 12, 13].

Therefore, this study aimed to assess the applicability of the ICD-MM guidelines by investigating the classification of maternal deaths in one country and across three countries. First, the cause of death as determined by the attending physician was compared to the assessment of the Surinamese MDR committee. Second, cases were shared with the national MDR committees from Jamaica and the Netherlands, and their assessments were compared to the findings of the Surinamese MDR committee. Following these findings, the classification difficulties are discussed, and recommendations for improving the ICD-MM guidelines’ applicability and international comparability of maternal mortality are provided.

## Methods

### Study design

A population-based reproductive age mortality survey (RAMoS) was conducted in 2015 to identify pregnancy-related deaths in Suriname between 2010 and 2014 [3]. A total of 89 possible maternal deaths were identified and reviewed by the national MDR committee of Suriname, Jamaica (both middle-income countries) and the Netherlands (high-income country).

### Settings

Suriname is an upper middle-income South American country on the Caribbean coast with 570,496 inhabitants in 2017 [14, 15]. Out of the approximately 10,000 births annually, 86% occur in five hospitals, 6% in primary care centers and the remaining 8% deliver at home or is not registered [16]. When death occurs, the attending physician in Suriname has an obligation to complete a death certificate documenting the causes and circumstances of the death. The Bureau of Public Health codes the cause of death using ICD-10 [3]. A national MDR committee was established to audit and classify the pregnancy-related cases. The committee consisted of specialists in obstetrics, internal medicine, midwifery and, on request, other specialists such as cardiologists, intensive care specialists and neurologists were invited. Classification was consensus-based, and according to the WHO ICD-MM guidelines [3].

Jamaica, a Caribbean island nation with 2.9 million inhabitants, is an upper middle-income country. Their MDR committee was established in 1998, and classified maternal deaths according to the ICD-MM [12]. Three members from this multidisciplinary committee (midwives, obstetricians, epidemiologists, public health practitioners, and pathologists), volunteered to review the Surinamese cases: a reproductive health epidemiologist and two obstetricians.

The Netherlands is a high-income country with 17.3 million inhabitants [17]. The MDR committee of the Dutch Society of Obstetricians and Gynecology, was established in 1981 and currently uses the ICD-MM guidelines for maternal death classification [13]. Seven committee members classified the pregnancy-related deaths of this study independently. In case of uncertainty or unclarity, cases were discussed with other members to achieve consensus on the final classification.

### Definitions

**Fig. 1:**
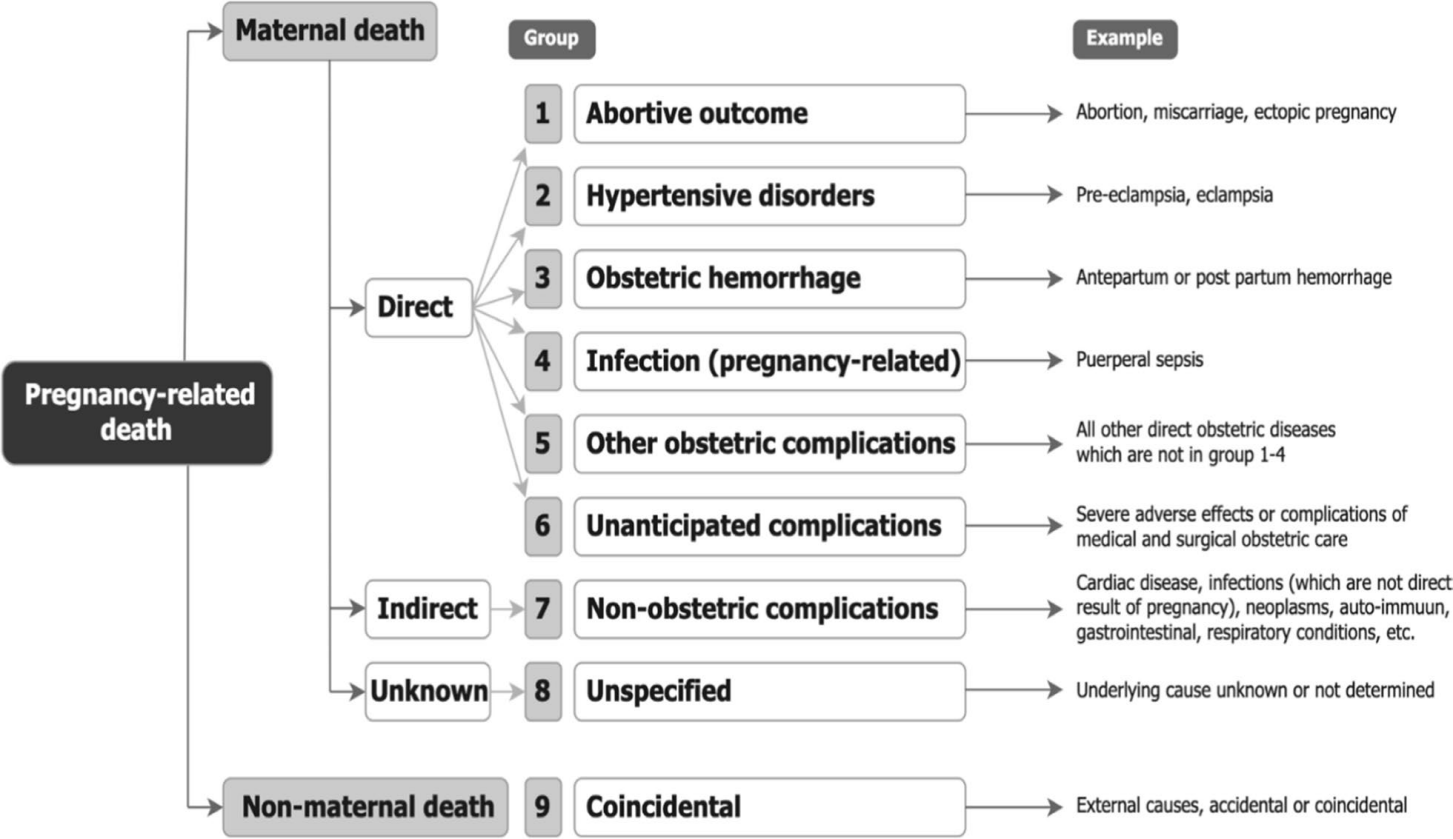
Groups of underlying causes of death during pregnancy, childbirth and the puerperium in mutually exclusive, totally inclusive groups [7]

Pregnancy-related deaths occur during pregnancy, delivery and puerperium. Maternal deaths are defined as those occurring during pregnancy or within 42 days of termination of pregnancy, irrespective of the duration and site of the pregnancy, where the cause of death is related to or aggravated by pregnancy or its management, not from coincidental or accidental causes [7]. Direct deaths are due to obstetric complications, while indirect deaths result from non-obstetric pre-existing diseases, or diseases developing during pregnancy, that is aggravated by the physiologic effects of pregnancy. If the underlying cause is unknown or undetermined, the death is classified as unspecified. Coincidental deaths are deaths that occur during pregnancy, childbirth and puerperium due to external causes that are not related to the pregnancy. Each pregnancy-related death can be assigned to one of nine groups: group 1–6 (direct deaths), group 7 (indirect deaths), group 8 (unspecified deaths), or group 9 (coincidental deaths) (Fig. 1) [7]. The underlying cause of death is the disease or condition that initiated the chain of events leading to death [6, 7].

### Data collection and analysis

Pregnancy-related deaths (n = 89) occurring in Suriname between 2010 and 2014 were identified by a Reproductive Age Mortality Survey (RAMoS) [3]. Medical files were summarized, and the underlying causes of death, as attributed by the attending physicians, were extracted from the available death certificate. All possible pregnancy-related deaths were audited by the Surinamese MDR committee and classified according to the ICD-MM [3]. In Suriname, we compared the underlying cause attributed by the attending physicians (documented on the death certificate or in the medical record) to the findings of the national MDR committee.

The Jamaican and Dutch MDR committees reviewed and classified the same 89 pregnancy-related deaths into maternal death or not, type of maternal death and one of the nine ICD-MM groups. Cases classified as not maternal by all three review teams were excluded from further analysis. The classification in type of death (direct, indirect and unspecified) and the WHO group of underlying cause were compared, using correlation analysis to assess agreement among the three review teams (IBM SPSS version 24.0; Armonk, New York, USA). The inter-rater reliability (IRR) was calculated by Fleiss kappa (for three raters). The kappa (κ) value range from − 1 to + 1, where 0 represents no agreement and one perfect agreement. Negative values indicate that the observed agreement is less than that expected from chance alone [18]. A κ below 0.2 indicates poor agreement and above 0.8 very good agreement. The overall value of kappa is the weighted average of the individual kappa value per category. A p-value < 0.05 only indicates that agreement between raters is significantly better than expected by chance [18, 19]. Discrepant cases were described to highlight sources of disagreement and facilitate further refinement of regional and global guidelines.

We performed two sensitivity analyses to evaluate the agreement across the MDR committees in type and underlying cause attribution. First, we excluded mortality cases that were not classified as maternal deaths by at least one MDR committee. Next, we assessed whether agreement on type and underlying cause attribution was better for maternal deaths with complete files.

## Results

Out of 89 pregnancy-related deaths, 53 (60%) medical files were complete, 14 (16%) were unavailable, and 22 were incomplete. The three MDR committees utilized all available information to analyze the 89 deaths.

### Classification in Suriname: attending physicians and the Surinamese MDR committee

In 42 (47%) of 89 pregnancy-related deaths the cause attributed by the attending physician and the MDR committee differed; seventeen had no underlying cause attributed by the attending physician, and in 25 cases, different causes were concluded by the MDR committee. Differences were mostly due to the mode of death or symptoms having been recorded as the underlying cause. Two autopsies had been performed, one on a possible late maternal death and another on a woman who had developed a pulmonary embolism after a placental abruption.

### Classification by the MDR committees of Suriname, Jamaica and the Netherlands

#### Maternal death classification

**Fig. 2:**
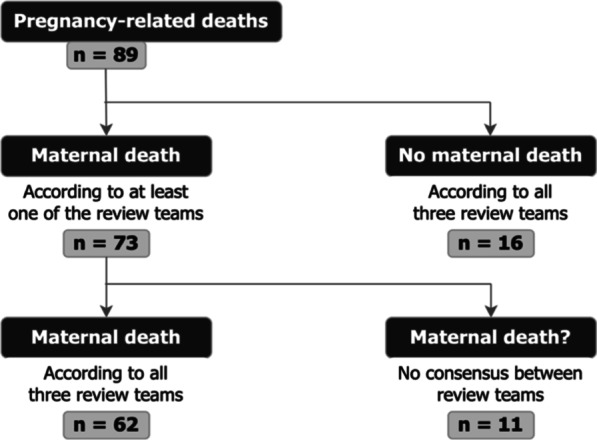
Flowchart of the pregnancy-related deaths classified by maternal death review (MDR) committees of Suriname, Jamaica and the Netherlands

The Surinamese MDR committee classified 65 deaths as maternal, the Jamaican MDR committee 70 and the Netherlands MDR committee 69. Based on 50,051 live births in the audited period, this corresponded with an MMR of 130, 140 and 138 per 100.000 live births, respectively. The three MDR committees agreed unanimously that 18% (n = 16/89) of the pregnancy-related deaths were not maternal deaths: 12 late maternal deaths, two coincidental deaths and two with negative pregnancy tests (Additional file 1). Exclusion of these cases resulted in a total of 73 cases, used for further analyses (Fig. 2). However, opinions differed in 15% (n = 11/73) of the cases (Table 1).

**Table 1:** Pregnancy-related deaths lacking consensus among maternal death review (MDR) committees whether to classify as maternal deaths

Case number	Gestational age	Case description; cause of death	Classified as a maternal death by MDR committee of		
			Suriname	Jamaica	Netherlands
*Doubt in classification of suicide in early pregnancy*					
1	Unknown	Gramoxone^a^ auto-intoxication	No	Yes	No
2	7 weeks	Gramoxone^a^ auto-intoxication	No	Yes	Yes
3	9 weeks	Gramoxone^a^ auto-intoxication	No	Yes	Yes
*Doubt regarding evidence of pregnancy*					
4	N/A	Following a curettage, chest pain and dyspnea developed. Curettage pathology report showed no evidence of pregnancy	No	No	Yes
5	N/A	Died at home from unknown cause. Verbal autopsy with family: early pregnancy. Examination: no fundal height palpable, but peripheral edema of both feet	Yes	Yes	No
6	N/A	Died in transit to hospital. Patient complained of abdominal pain, vaginal blood loss and chest pain. Verbal autopsy with family: could be pregnant	No	Yes	Yes
*Doubt whether the death was maternal or coincidental*					
7	25 weeks	Severe burn wounds after explosion	No	No	Yes
8	29 weeks	Sepsis, meningoencephalitis/cerebral abscess	Yes	Yes	No
9	34 days postpartum	Sepsis with symptoms of high fever and diarrhea	Yes	No	Yes
10	35 days postpartum	Normal delivery. Cause of death unknown	No	Yes	Yes
*Doubt in classification of maternal death of a local resident in another country*					
11	29 weeks	Admitted in a Surinamese hospital with a severe sickle cell crisis. Ten days after discharge she died in neighboring French Guyana	No	Yes	No

^a^Gramoxone is the tradename of paraquat, a contact herbicide, highly toxic to humans

#### Classification into type of maternal deaths (direct, indirect and unspecified)

Of the 73 cases considered as maternal deaths by at least one MDR committee, classification into type of maternal death differed for 31 (42%) cases. The overall kappa was 0.53 (95% CI 0.44—0.62); p < 0.001 and was only fair for the unspecified category (κ = 0.29 (95% CI 0.16 – 0.43); p < 0.001) (Table 2). The Netherlands committee (19%, n = 14/73) classified more cases as unspecified compared to Surinamese (4%, n = 3/73) and Jamaican committees (7%, n = 5/73) (Table 2 and Additional file 2). Agreement between the MDR committees of Suriname and Jamaica (κ = 0.69 (95% CI 0.53–0.86); p < 0.001) was higher than between the committees of Suriname and the Netherlands (κ = 0.48 (95% CI 0.32 – 0.63); p < 0.001) (Table 2). Out of 41 maternal deaths classified as direct by the Surinamese committee, the Jamaican committee classified five cases differently (four indirect, one unspecified), while the Dutch committee classified ten cases otherwise (three indirect, seven unspecified).

**Table 2:** Agreement among Maternal Death Review (MDR) committees in classification into the type of maternal death

Type of maternal death n = 73 (100%)	MDR committees		
	Suriname n (%)	Jamaica n (%)	The Netherlands n (%)
Direct	41 (56)	41 (56)	36 (49)
Indirect	21 (29)	24 (33)	19 (26)
Unspecified	3 (4)	5 (7)	14 (19)
Not Maternal	8 (11)	3 (4)	4 (6)
Kappa = 0.53 (95% CI 0.44—0.62); p < 0.001			

	Surinamese MDR committee					
	Type of maternal death n = 73	Direct	Indirect	Unspecified	Not Maternal	*Total Jamaica*
	Direct	36	1	0	4	*41*
	Indirect	4	19	0	1	*24*
**Jamaican MDR committee**	Unspecified	1	0	3	1	*5*
	Not Maternal	0	1	0	2	*3*
	*Total Suriname*	*41*	*21*	*3*	*8*	*73*
Kappa = 0.69 (95% CI 0.53 - 0.86); p < 0.001						
	Type of maternal death n = 73	Direct	Indirect	Unspecified	Not Maternal	*Total Netherlands*
	Direct	31	3	0	2	*36*
	Indirect	3	14	0	2	*19*
**The Netherlands MDR committee**	Unspecified	7	3	2	2	*14*
	Not Maternal	0	1	1	2	*4*
	*Total Suriname*	*41*	*21*	*3*	*8*	*73*
Kappa = 0.48 (98% CI 0.32 - 0.63); p < 0.001						

#### Classification into WHO ICD-MM groups of underlying causes

Table 3 compares the underlying causes of maternal deaths according to the nine ICD-MM groups as classified by the three MDR committees. Table 4 summarizes levels of agreement between the three MDR committees for each ICD-MM underlying cause group. The overall kappa was 0.52 (95% CI 0.47–0.58); p < 0.001, with the highest agreement for abortive outcomes (κ = 0.85) and obstetric hemorrhage (κ = 0.74) and the lowest for the unspecified (κ = 0.29) and other direct causes (κ = 0.32).

**Table 3:** Classification of maternal deaths underlying causes according to the ICD-MM by the three maternal death review (MDR) committees

Underlying cause of maternal death n = 73 (100%)	MDR Committees		
	Suriname n (%)	Jamaica n (%)	The Netherlands n (%)
**Direct**			
1. Abortive outcomes	2 (3)	3 (4)	2 (3)
2. Hypertensive disorders	7 (10)	11 (15)	6 (8)
3. Obstetric hemorrhage	13 (18)	16 (22)	14 (19)
4. Infection (pregnancy-related)	6 (8)	2 (3)	5 (7)
5. Other obstetric complications	13 (18)	9 (12)	9 (12)
6. Unanticipated complications	-	-	-
**Indirect**			
7. Non-obstetric complications	21 (29)	24 (33)	19 (26)
**Unknown**			
8. Unspecified	3 (4)	5 (7)	14 (19)
**Not maternal**			
9. Coincidental	8 (11)	3 (4)	4 (6)

**Table 4:** Level of agreement of underlying causes according to WHO ICD-MM by maternal death review (MDR) committees of Suriname, Jamaica and the Netherlands

Levels of agreement	Suriname, Jamaica and Dutch MDR committee	Surinamese and Jamaican MDR committee	Surinamese and Dutch MDR committee
Almost perfect Kappa ≥ 0.81	Abortive outcomes	-	Abortive outcomes
Substantial Kappa 0.61–0.80	Obstetric hemorrhage Indirect	Abortive outcomes Hypertensive disorders Obstetric hemorrhage Indirect Unspecified	Obstetric hemorrhage
Moderate Kappa 0.41–0.60	Hypertensive disorders Obstetric infection	Obstetric infection	Hypertensive disorders Obstetric infection Indirect
Fair Kappa 0.21–0.40	Unspecified Other direct obstetric	Other direct obstetric	Other direct obstetric
Poor/Slight Kappa < 0.20	-	-	Unspecified

Agreement between the Surinamese and Jamaican MDR committees was higher (overall κ = 0.63; 95% CI 0.53–0.73); p < 0.001 than between the Surinamese and Dutch committees (overall κ = 0.49; 95% CI 0.39–0.59); p < 0.001. The lowest agreement between the Surinamese and the Jamaican MDR committees was for other direct obstetric causes (κ = 0.36) and highest for obstetric hemorrhage (κ = 0.79) and indirect deaths (κ = 0.78). Agreement was poor between the Surinamese and the Dutch MDR committee for unspecified (κ = 0.14) and other direct deaths (κ = 0.25).

The sensitivity analyses on the agreement between the MDR committees in type and underlying causes was performed on 62 cases (by excluding all mortality cases that were assessed as being not maternal). These showed slightly better overall agreement for classification in type of maternal death (κ = 0.61 vs 0.53) and underlying cause (κ = 0.58 vs 0.52) compared to the primary analysis (Additional file 3). Fifty-three maternal death cases had complete files. Analysis of only the cases with complete files also showed better overall agreement for classification in type of maternal death (κ = 0.69 vs 0.53), and underlying cause (κ = 0.58 vs 0.52) than the primary analysis.

Evaluation of the level of agreement for the ICD-MM underlying cause among the MDR committees showed better agreement between Suriname and Jamaica (κ = 0.69 vs 0.66) than between Suriname and the Netherlands (κ = 0.54 vs 0.53) when applied to the 62 maternal deaths, as well as when applied to the 53 complete files respectively (Additional file 3).

### Cycle of morbid events leading to death and classification by countries’ MDR committees.

**Fig. 3:**
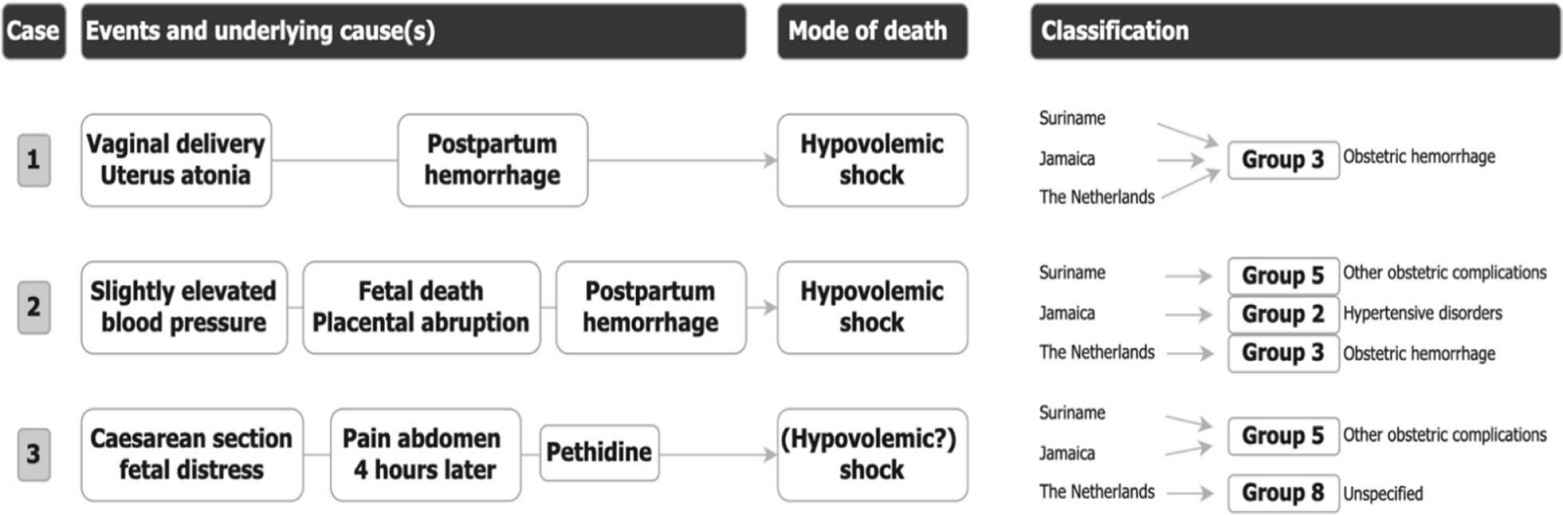
Maternal death classification difficulties in simple and complex chain of events

Consensus among the Surinamese, Jamaican and Dutch committees was fair for the other direct obstetric causes, with three cases identically classified (two presumed amniotic fluid embolisms, one suicide at 24 weeks) (Table 4 and Additional file 4). The cases with discrepancies in groups of underlying cause were characterized by either multiple comorbidities and longer chain of events or rapidly evolving death without opportunities for additional diagnostic evaluation (Fig. 3).

## Discussion

This study explored consistency in classifying pregnancy-related deaths in Suriname at two levels. First, underlying cause attribution by the attending physicians, and the Surinamese MDR committee was compared; conclusions differed in 47% of cases. Second, the classification of three national MDR committees of Suriname, Jamaica and the Netherlands were compared applying the WHO ICD-MM guidelines to the same cases. There was 15% disagreement among these committees on whether selected pregnancy-related deaths met the criteria to be defined as maternal deaths. They achieved moderate agreement (k = 0.53) on classifying cases as direct, indirect or unspecified, with greater consensus between the Surinamese and Jamaican MDR committees (k = 0.69) than the Surinamese and Netherlands MDR committees (k = 0.48). The MDR committee of the Netherlands, a high-income country, classified more deaths as unspecified than those from the middle-income countries of Suriname and Jamaica. There was higher concurrence among the three MDR committees in underlying cause attribution to abortive outcomes, obstetric hemorrhage and indirect maternal deaths, but only fair agreement on a mix of cases (other direct obstetric causes and unspecified).

The large difference (47%) in underlying cause attribution for maternal death between the attending physicians and the Surinamese MDR committee is not unusual. Similar differences were also seen in Malawi, where poor agreement between healthcare providers and the research team on maternal death classification was reported [20]. Another study found a 40% difference in underlying cause attribution in a multi-country survey that compared health provider findings with external reviewers among Low- and Middle-Income Countries (LMIC) [6]. The abovementioned examples illustrate the importance of multidisciplinary case discussion and consensus-based underlying cause attribution.

Besides inconsistent underlying cause attribution, poor coding of pregnancy-related deaths, misidentification, or misclassification can result in inadequate certification and is associated with underreporting [2, 12, 21]. Due to underreporting, vital statistics could miss at least 50% of the maternal deaths [22]. Hence, since maternal death certificates are also completed by non-obstetricians (e.g. in the rural interior or when indirect maternal deaths occurred), all clinicians would benefit from training to correctly complete death certificates.

The MDR committees in our study encountered specific challenges for which no clear guidance was available from the ICD-MM guidelines. These included (1) determining the fact of pregnancy with limited evidence; (2) inclusion of deaths from suicide, especially in early pregnancy and (3) whether and how to count maternal deaths outside the country of residence. It is unclear what the minimally acceptable evidence of pregnancy should be without medical confirmation and under which circumstances information from verbal autopsy alone could be used to confirm pregnancy. While the ICD-MM classifies suicide during pregnancy and puerperium as a direct maternal death, this is clearer for puerperal psychosis and postpartum depression than for events early in pregnancy [7]. The trigger for suicide may be social/circumstantial (partner rejection, domestic violence, unintended pregnancy), rather than clinical (pre-existing mental disorder or hormonal changes impacting mental health) [23, 24]. In addition, the ICD-MM guidelines do not elaborate on how to classify maternal deaths from suicide (direct vs indirect) when underlying mental disorders existed [23]. Finally, opinions differed in this study on the inclusion of a resident who had been under local care but died in another country. As no global guidance exists on whether to count such events in the country where the women dies or the country of residence, there is a chance that these cases are not reported at all (excluded in the country where she died and not reported in the country where she lived). Since all births are included in the national birth registry (denominator), we suggest including the mother also in the country where she died (numerator). Importantly, in these situations information is ideally exchanged between countries to facilitate local reporting and sharing of “lessons to be learned”.

Consensus between the MDR committees of Suriname and Jamaica was higher than between those of Suriname and the Netherlands. The cases the Dutch committee considered unspecified but were assigned other diagnoses by the other committees had limited information on the disease course, and lacked confirmatory diagnostic tests such as laboratory results, ultrasounds, Computed Tomography (CT) or Magnetic Resonance Imaging (MRI) scans compared to the cases with more agreement. Advanced diagnostics were often unavailable due to financial or logistic constraints, such as the minimal laboratory capacity in the rural interior areas. In these cases, the MDR committees in LIMC must often rely on clinical judgement to make a diagnosis. Practicing medicine with greater uncertainty regarding diagnosis and treatment outcomes and fewer possibilities to provide evidence-based care is more commonplace in LMIC and possibly explains the more consistent results between the MDR committees of the two middle-income countries.

Classification into type of maternal death (direct, indirect and unspecified) differed in 42% of cases, only achieving moderate agreement among the three MDR committees. Dividing maternal deaths into direct and indirect conditions is pragmatic as preventive programs to avert direct deaths differ from indirect deaths [25]. However, this division has been questioned by the MDR committees in the United Kingdom (UK) and the Netherlands, especially for women with concurrent direct and indirect comorbidities [26]. In both middle and high-income countries, several pre-existing conditions such as obesity, diabetes mellitus, and hypertensive diseases are increasing and the risk of pregnant women to develop direct and indirect complications of pregnancy (e.g. postpartum hemorrhage, eclampsia, cardiovascular diseases) [26–28]. This coexistence of multiple conditions in an individual is known as multimorbidity and is one of the challenges of modern medicine [29, 30]. These conditions obfuscate the strict demarcation between direct and indirect deaths and reduce their relevance. Instead, adding multimorbidity categories, such as (non)communicable diseases and (pre-existing) mental disorders to the ICD-MM guidelines would be more pertinent.

We conducted a sensitivity analysis to explore whether consensus improved with the exclusion of (1) cases without consensus among the MDR committees in the classification as maternal deaths, and (2) cases with incomplete information. As expected, the exclusion of the cases with uncertainty improved the level of agreement. These exclusions strengthened the consensus that already existed between the Surinamese and Jamaican MDR committees. However, since differences are small, these analyses suggest that, even with limited information, MDR committees can reach reliable conclusions on the probable types and underlying causes of maternal deaths.

Our data showed that when the cycle of events leading to death had fewer incidents (Fig. 3), underlying cause attribution was more straightforward (as with abortion-related and obstetric hemorrhage). Selecting the initiating event from a chain of multiple events is more difficult in complex cases, resulting in a discrepancy in underlying cause classification in our study. Two high-income countries, the United Kingdom (UK) and the Netherlands, also reported such differences in underlying cause attribution [10]. Their MDR committees discussed selected cases where disagreement was expected during a meeting attended by most members of both committees*.* While the Netherlands classified a death by the primary underlying pathology, the UK more pragmatically focused on the acute fatal complication [10]. They suggested that decision-making may be guided by what best informs local practice in the absence of global guidance. However, this approach could result in heterogeneity and complicates comparison among countries.

Reliable underlying cause attribution may be improved by combining clinical data with autopsy findings [31, 32]. However, autopsy for maternal death is seldom performed in low resource countries such as Suriname, where only two cases were investigated [3]. It may be useful to revisit verbal autopsy techniques to improve collection and interpretation of information on signs, symptoms and risk factors [33]. Another possible option is the minimally invasive autopsy. This includes collection of blood, cerebrospinal fluid and tissue samples for histologic and microbiologic analysis [34]. This option could be explored to assist in identifying the underlying causes of maternal death.

### Strengths and limitations

This study’s strength is its unique comparison of the classification of the same cases by physicians and (inter)national MDR committees from three different settings applying the WHO ICD-MM guidelines. Limitations include difficulties in interpreting cases with limited information and, possibly, by a high-income country being unfamiliar with the different contexts of LMIC. The inter-rater reliability should be carefully interpreted as the overall kappa may not be reliable for rare observations, such as group 1 (abortive outcomes) and group 4 (pregnancy-related infections).

## Conclusion and recommendations

This is the first study comparing audit and ICD-MM classification of the same maternal deaths by MDR committees of different countries, revealing the difficulties and challenges. Accurately completing the death certificate, training in performing audits and applying the WHO ICD-MM guidelines to code and classify the death should be encouraged [12, 17]. We suggest that the WHO guidelines should elaborate more on the following aspects:

1. Clearly define and describe how to classify suicide during (early) pregnancy or puerperium.
2. Provide guidance on the minimal acceptable evidence of early pregnancy in the absence of objective clinical evidence (e.g. a pregnancy test), and specify on the use of information obtained through verbal autopsy.
3. Specify where maternal deaths of citizens who die outside of their country of residence should be counted to ensure that all maternal deaths globally are counted.
4. Discuss the relevance of classification in direct or indirect causes and the addition of classification in multimorbidity categories.
5. Provide guidance on selecting the underlying causes when concurrent comorbid direct and indirect conditions exist, or multiple direct complications co-occur.

## Supplementary Information

**Additional file 1.** Case description of the 2010–2014 pregnancy-related deaths in Suriname classified as “no maternal death” by all three MDR committees.

**Additional file 2.** Case description of the 2010–2014 maternal deaths of Suriname classified as “unspecified”.

**Additional file 3.** Sensitivity analysis for type of maternal death and WHO ICD-MM group of underlying causes.

**Additional file 4.** Case description of 2010–2014 maternal deaths in Suriname classified as “other direct obstetric causes”.

## Data Availability

The datasets used and analyzed for this study are available from the corresponding author upon request.

## Abbreviations

WHO: World Health Organization
ICD-MM: International Classification of Diseases-Maternal Mortality
MDR: Maternal Death Review
LIMC: Low and Middle-Income countries
UK: United Kingdom
MRI: Magnetic Resonance Imaging
CT: Computed Tomography
