# Supplementary material for: Classifying maternal deaths in Suriname using WHO ICD-MM: different interpretation by Physicians, National and International Maternal Death Review Committees
Source: Reprod Health. 2021 Feb 19;18:46. doi: 10.1186/s12978-020-01051-1 (PMC7893967; doi:10.1186/s12978-020-01051-1)
Supplement: Supplementary file 1 — Additional file 1. Case description of the 2010–2014 pregnancy-related deaths in Suriname classified as “no maternal death” by all three MDR committees. [file 12978_2020_1051_MOESM1_ESM.docx]

**Additional file 1. Case description of the 2010-2014 pregnancy-related deaths in Suriname classified as “no maternal death” by all three MDR committees**

| **Case number** | **Gestational age** | **Description** |
| --- | --- | --- |
| **Pregnancy test negative** | | |
| 1 | Last menstruation 4 weeks ago | Pain in the belly, severe anemia, pregnancy test negative |
| 2 | According to husband pregnant | Pain in the belly, pregnancy test negative, human chorionic gonadotrophins (HCG) < 1 |
| **Coincidental deaths** | | |
| 3 | 24 weeks | Hemorrhagic shock following impact trauma |
| 4 | 25 weeks | Trauma capitis and pneumo-sepsis |
| **Late maternal deaths** | | |
| 5 | 8 weeks postpartum | Tachypnoe, diarrhea, vomiting |
| 6 | 9 weeks after caesarean | Diarrhea, fever, vomiting, acute respiratory distress |
| 7 | 10 weeks postpartum | Pre-eclampsia. Developed meningitis/encephalitis |
| 8 | 11 weeks postpartum | Breast cancer |
| 9 | 11 weeks postpartum | Cervical cancer |
| 10 | 12 weeks postpartum | Peripartum cardiomyopathy |
| 11 | 16 weeks after early pregnancy | Probably early pregnancy (human chorionic gonadotrophins (HCG) 181), after 16 weeks pulmonary embolus, autopsy showed no pregnancy |
| 12 | 16 weeks after caesarean | Pregnancy complicated by pre-eclampsia. Died at home suddenly |
| 13 | 19 weeks postpartum | Esophageal cancer |
| 14 | 24 weeks postpartum | Delivered premature (at 35 weeks), lupus erythematosus and sickle cell anemia. |
| 15 | 24 weeks postpartum | Peripartum cardiomyopathy |
| 16 | 41 weeks after caesarean | Laparotomy due to ileus, many adhesions |
