# Supplementary material for: Classifying maternal deaths in Suriname using WHO ICD-MM: different interpretation by Physicians, National and International Maternal Death Review Committees
Source: Reprod Health. 2021 Feb 19;18:46. doi: 10.1186/s12978-020-01051-1 (PMC7893967; doi:10.1186/s12978-020-01051-1)
Supplement: Supplementary file 2 — Additional file 2. Case description of the 2010–2014 maternal deaths of Suriname classified as “unspecified”. [file 12978_2020_1051_MOESM2_ESM.docx]

**Additional file 2. Case description of the 2010-2014 maternal deaths of Suriname classified as “unspecified”**

| Case | Gestation | Case description | Classification by countries MDR Committees | | |
| --- | --- | --- | --- | --- | --- |
|  | | | **Suriname** | **Jamaica** | **Netherlands** |
| **1** | 9 months pregnant | Came from the interior, died in the car and was immediately transferred to the mortuary. | Unspecified | Unspecified | Unspecified |
| **2** | 23 weeks according to family | Died at home in the interior. Complained of headache and dizziness 3 months before. | Unspecified | Unspecified | Unspecified |
| **3** | Verbal autopsy: early pregnancy | Died at home from unknown cause. Verbal autopsy with family: early pregnancy. Examination: no fundal height palpable, but peripheral edema of both feet. | Unspecified | Unspecified | No  Maternal death |
| **4** | 35 days  postpartum | Uncomplicated childbirth. Died at home 35 days later. No further information available. | No  Maternal death | Unspecified | Unspecified |
| **5** | 3 days  postpartum | Uncomplicated childbirth. Died 3 days later with pain in legs and belly. No further information available. | Other  Direct causes | Unspecified | Other  Direct causes |
| **6** | Early pregnancy | Died before reaching hospital. Had abdominal pain, vaginal blood loss and chest pain. Verbal autopsy with family: could be pregnant | No  Maternal death | Direct | Unspecified |
| **7** | 25 weeks | Severe hypertension and severe dyspnea followed by respiratory arrest | Direct | Direct | Unspecified |
| **8** | 35 weeks | Grande multiparous, breech delivery in interior, retained placenta, died during transport to city. Blood loss not recorded. | Direct | Direct | Unspecified |
| **9** | 37 weeks | Obstructed labor, died in interior 3 hours after full dilation and ruptured membranes. | Direct | Direct | Unspecified |
| **10** | 1 week postpartum | Two previous caesareans. Delivery of dead child at 27 weeks, curettage postpartum. One week later died at ER with normal vitals, normal laboratory results. | Direct | Direct | Unspecified |
| **11** | 5 hours after caesarean | Caesarean for fetal distress at 41 weeks, stillbirth born. Post-caesarean hypotensive and found dead an hour later. | Direct | Direct | Unspecified |
| **12** | 40 weeks | Died suddenly during delivery after rupture of membranes. | Direct | Direct | Unspecified |
| **13** | +/- 27 weeks | Found in vomit, unconscious with fever and high pulse and died. Abnormal kidney and liver enzymes. | Direct | Indirect | Unspecified |
| **14** | 35 weeks | Atypical pneumonia, respiratory insufficiency and died after one week. | Indirect | Indirect | Unspecified |
| **15** | 2 weeks postpartum | AIDS with heart failure and sepsis. No medical file available. | Indirect | Indirect | Unspecified |
| **16** | +/- 30 weeks | Known with an unknown illness before pregnancy. Vomits and probably had an exacerbation of disease. Did not seek medical help and died in interior. | Indirect | Indirect | Unspecified |
