# Supplementary material for: Classifying maternal deaths in Suriname using WHO ICD-MM: different interpretation by Physicians, National and International Maternal Death Review Committees
Source: Reprod Health. 2021 Feb 19;18:46. doi: 10.1186/s12978-020-01051-1 (PMC7893967; doi:10.1186/s12978-020-01051-1)
Supplement: Supplementary file 3 — Additional file 3. Sensitivity analysis for type of maternal death and WHO ICD-MM group of underlying causes. [file 12978_2020_1051_MOESM3_ESM.docx]

**Additional file 3. Sensitivity analysis for type of maternal death and WHO ICD-MM group of underlying causes**

| **Type of maternal death** | | | |
| --- | --- | --- | --- |
| **Agreement among MDR committees [κ (95% CI)]** | | | |
|  | **Overall** | **Suriname - Jamaica** | **Suriname - the Netherlands** |
| All files (n=73) | 0.53 (0.44-0.62) | 0.69 (0.53-0.86) | 0.48 (0.32-0.63) |
| Only cases with consensus on classification as maternal death (n= 62) | 0.61 (0.49-0.72) | 0.80 (0.58-1.0) | 0.52 (0.33-0.72) |
| Complete files only (n=53) | 0.69 (0.58-0.79) | 0.80 (0.60-0.99) | 0.64 (0.46-0.82) |

| **WHO ICD-MM group of underlying causes** | | | |
| --- | --- | --- | --- |
| **Agreement among MDR committees [κ (95% CI)]** | | | |
|  | **Overall** | **Suriname - Jamaica** | **Suriname - the Netherlands** |
| All files (n=73) | 0.52 (0.47–0.58) | 0.63 (0.53–0.73) | 0.49 (0.39–0.59) |
| Only cases with consensus on classification as maternal death (n= 62) | 0.58 (0.52–0.65) | 0.69 (0.57–0.81) | 0.54 (0.43–0.66) |
| Complete files only (n=53) | 0.58 (0.51-0.65) | 0.66 (0.54-0.78) | 0.53 (0.42-0.64) |
