# Supplementary material for: Classifying maternal deaths in Suriname using WHO ICD-MM: different interpretation by Physicians, National and International Maternal Death Review Committees
Source: Reprod Health. 2021 Feb 19;18:46. doi: 10.1186/s12978-020-01051-1 (PMC7893967; doi:10.1186/s12978-020-01051-1)
Supplement: Supplementary file 4 — Additional file 4. Case description of 2010–2014 maternal deaths in Suriname classified as “other direct obstetric causes”. [file 12978_2020_1051_MOESM4_ESM.docx]

**Additional file 4. Case description of the 2010-2014 maternal deaths of Suriname classified as “other direct obstetric causes”**

| Case | Gestation | Case description | Classification by countries MDR Committees | | |
| --- | --- | --- | --- | --- | --- |
|  | | | **Suriname** | **Jamaica** | **Netherlands** |
| **1** | 39 weeks | Collapsed during delivery, amniotic fluid embolism | Other Direct Causes | Other Direct  Causes | Other Direct  Causes |
| **2** | 41weeks | Collapsed during delivery, amniotic fluid embolism | Other Direct Causes | Other Direct  Causes | Other Direct  Causes |
| **3** | 24 weeks | Suicide by autointoxication | Other Direct Causes | Other Direct  Causes | Other Direct  Causes |
| **4** | 0 days postpartum | Hypertension, followed by a placental abruption complicated by massive blood loss after childbirth. | Other Direct Causes | Hypertensive | Hemorrhage |
| **5** | 30 days postpartum | Pre-eclampsia complicated by intra-cranial cerebral bleeding postpartum. | Other Direct Causes | Hypertensive | Other Direct  Causes |
| **6** | 27 weeks | Home delivery with stillbirth. Normotensive with headache and vomiting. Died one week later. | Other Direct Causes | Hypertensive | Unspecified |
| **7** | 3 days postpartum | Lupus and hypertension with thrombocytopenia complicated by massive blood loss. | Other Direct Causes | Hemorrhage | Hemorrhage |
| **8** | 0 days postpartum | Hypertension and abruption placentae. Uterine torsion, hysterectomy and massive blood loss. | Other Direct Causes | Hemorrhage | Hypertensive |
| **9** | 37 weeks | Obstructed labor, died in interior 3 hours after full dilation and ruptured membranes. | Other Direct Causes | Hemorrhage | Unspecified |
| **10** | 11 days postpartum | Severe pre-eclampsia, stillbirth of one of twins, caesarean section, followed by severe hematemesis, coagulation disorder and respiratory insufficiency. | Other Direct Causes | Indirect | Indirect |
| **11** | 41 days postpartum | Pulmonary hypertension with respiratory insufficiency six weeks after childbirth. No pulmonary embolism. | Other Direct Causes | Indirect | Indirect |
| **12** | 40 weeks | Died instantly during labor when the membranes ruptured | Other Direct Causes | Other Direct  Causes | Unspecified |
| **13** | 0 days postpartum | Found dead in her bed five hours after caesarean section | Other Direct Causes | Other Direct  Causes | Unspecified |
| **14** | 34 weeks | Immune thrombocytopenic purpura. Pulmonary bleeding and respiratory insufficiency. | Indirect | Other Direct  Causes | Indirect |
| **15** | Early pregnancy | Suicide by autointoxication | No maternal death | Other Direct  Causes | Other Direct  Causes |
| **16** | Early pregnancy | Suicide by autointoxication | No maternal death | Other Direct  Causes | No maternal |
| **17** | 10 weeks | Suicide by autointoxication | No maternal death | Other Direct  Causes | Indirect |
| **18** | 1 day postpartum | Caesarean section complicated by respiratory insufficiency in woman with a previously unknown severe mitral valve stenosis | Indirect | Indirect | Other Direct  Causes |
| **19** | 0 days postpartum | Severe pre-eclampsia with pulmonary edema. Fit after childbirth and cardiac arrest | Hypertensive | Hypertensive | Other Direct  Causes |
| **20** | 0 days postpartum | Hypovolemic shock due to uterus rupture | Hemorrhage | Hemorrhage | Other Direct  Causes |
| **21** | Early pregnancy | Died before hospital admission. Following curettage, chest pain and dyspnea developed. Curettage pathology report showed no evidence of pregnancy. | No maternal death | No maternal death | Other Direct  Causes |
